# Memory Complaints Associated with Seeking Clinical Care

**DOI:** 10.1155/2012/725329

**Published:** 2012-03-24

**Authors:** Carolina Pires, Dina Silva, João Maroco, Sandra Ginó, Tiago Mendes, Ben A. Schmand, Manuela Guerreiro, Alexandre de Mendonça

**Affiliations:** ^1^Dementia Clinics, Institute of Molecular Medicine and Faculty of Medicine, University of Lisbon, Avenue Prof. Egas Moniz, 1649-028 Lisbon, Portugal; ^2^Superior Institute of Applied Psychology, Rua Jardim do Tabaco, 34, 1149-041 Lisbon, Portugal; ^3^Santa Casa da Misericórdia de Lisboa, Largo Trindade Coelho, 1200-470 Lisbon, Portugal; ^4^Psychiatry Department, Santa Maria Hospital, Avenue Prof. Egas Moniz, 1649-035 Lisbon, Portugal; ^5^Faculty of Social and Behavioural Sciences, University of Amsterdam, 1018 vz Amsterdam, The Netherlands; ^6^Laboratory of Language, Institute of Molecular Medicine and Faculty of Medicine, University of Lisbon, Avenue Prof. Egas Moniz, 1649-028 Lisbon, Portugal; ^7^Laboratory of Neurosciences, Institute of Molecular Medicine and Faculty of Medicine, University of Lisbon, Avenue Prof. Egas Moniz, 1649-028 Lisbon, Portugal

## Abstract

Diagnosis of mild cognitive impairment relies on the presence of memory complaints. However, memory complaints are very frequent in healthy people. The objective of this study was to determine the severity and type of memory difficulties presented by elderly patients who seek for clinical help, as compared to the memory difficulties reported by subjects in the community. Assessment of subjective memory complaints was done with the subjective memory complaints scale (SMC). The mini-mental state examination was used for general cognitive evaluation and the geriatric depression scale for the assessment of depressive symptoms. Eight-hundred and seventy-one nondemented subjects older than 50 years were included. Participants in the clinical setting had a higher total SMC score (10.3 ± 4.2) than those in the community (5.1 ± 3.0). Item 3 of the SMC, *Do you ever forget names of family members or friends? *contributed significantly more to the variance of the total SMC score in the clinical sample (18%) as compared to the community sample (11%). Forgetting names of family members or friends plays an important role in subjective memory complaints in the clinical setting. This symptom is possibly perceived as particularly worrisome and likely drives people to seek for clinical help.

## 1. Introduction

Since the original description of the disease by Alois Alzheimer [1], memory difficulties are considered the initial and the most prominent and typical symptom of Alzheimer's disease. More recently, the detection of elderly subjects with mild cognitive impairment and a high risk of progression to Alzheimer's disease also relies on the presence of memory complaints [2], and, in proposed revised criteria for prodromal AD, like the Dubois criteria [3], the report by patients or informants of memory decline remains part of the core diagnostic features. Memory complaints thus represent an important symptom in clinical practice. 

On the other hand, when specifically asked for, people in the community frequently report memory difficulties. In fact, not only elderly but also young subjects may have an unfavourable opinion about their memory capabilities [4]. Using a formal scale, the subjective memory complaints scale (SMC; [5]), as much as 75.9% of people report at least minor complaints when answering to the first general question *Do you have any complaints concerning your memory*? (SMC1) [4]. The significance and clinical implications of the frequent report of memory difficulties in the community setting are not clear. In a meta-analysis, the presence of memory complaints was more frequent in cognitively impaired than in cognitively normal elderly subjects [6]. A systematic review concluded that the subjective memory complaints appear to be associated with depressive symptoms and personality traits and may predict future cognitive decline [7]. A recent study found that self perceived memory complaints are an independent predictor of dementia [8].

In the present study, we analysed the severity and type of memory difficulties presented by patients who look for medical help in a memory clinic or hospital outpatient setting, as compared to the memory difficulties that subjects in the community report when specifically asked for. The same instrument, the subjective memory complaints scale, was applied in both samples. We tested the hypothesis that some types of memory complaints would be selectively reported by subjects in the clinical setting. The objective was to identify the memory complaints most prone to raise concern from the patients and their families and bring them to clinical care.

## 2. Methods

### 2.1. Patients

Subjects with cognitive complaints older than 50 years referred for neuropsychological evaluation at the Laboratory of Language Studies, Santa Maria Hospital, and a Memory Clinic, both in Lisbon.

### 2.2. Controls

Controls were volunteers older than 50 years attending a health itinerant unit that aims to screen and promote general health, a blood donor centre, a leisure centre for retired people, and a senior citizens college and university, all in the area of Lisbon.

### 2.3. Exclusion Criteria

1. presence of dementia (criteria of the American Psychiatric Association, DSM-IV-TR [9]), or MMSE score below the education-adjusted cutoff,
2. neurological disorders (stroke, tumors, significant head trauma, and epilepsy), psychiatric conditions (including major depression), or uncontrolled medical illness (hypertension, metabolic, endocrine, toxic, and infectious diseases) able to interfere with cognition,
3. psychoactive medications with possible influence on cognition,
4. chronic alcohol or drug abuse,
5. sensory deficits likely to interfere with assessment, and
6. nonnative Portuguese speakers.

All participants gave their informed consent. The present study was approved by the local ethics committee.

### 2.4. Procedures

A semi-structured interview recorded clinical information, present and past medical conditions, psychiatric and neurological history, medication, social and familial status.

### 2.5. General Cognitive Assessment

The mini-mental state examination (MMSE; [10, 11]) was used for a general cognitive assessment. Participants with MMSE below education-adjusted values for the Portuguese population were excluded (<23 for less than 11 years of education, <28 for more than 11 years of education; [11]).

### 2.6. Assessment of Subjective Memory Complaints

Participants were assessed with the subjective memory complaints scale (SMC; [5, 12]). They were required to answer 10 individual items concerning difficulties in daily-life memory tasks, with total scores ranging from 0 (absence of complaints) to 21 (maximal complaints score). These items are considered representative of common memory complaints [5]. The SMC was always applied at the end of the clinical interview.

### 2.7. Assessment of Depression

For the assessment of depressive symptoms the geriatric depression scale (GDS; [13, 14]) was used. The version with 15 items was chosen.

### 2.8. Statistical Analysis

Statistical analyses were performed using IBM SPSS Statistics 19 for Windows (SPSS Inc., an IBM Company, Chicago, IL, USA). A probability value less or equal to 0.05 was assumed as statistically significant. Differences in the total SMC scores among the different community settings were tested with one-way ANOVA. Comparison of demographic and neuropsychological data between the participants in the community and in the clinical setting was done using Student's *t* test on quantitative variables and the Fisher's exact test on the nominal variable. Comparison of the SMC items and the SMC total score between participants in the community and in the clinical setting was performed with the Mann-Whitney *U* test. *Eta-*squared values were calculated for the individual SMC items to explain the total SMC score with ANCOVA, controlling for depression score and education years. The 95% confidence intervals for *eta*-squared values were obtained by nonparametric Bootstrap sampling (*k* = 1000) using the boot library in the R system software (v. 12.2.1, R Development Core Team).

## 3. Results

Eight-hundred and seventy-one nondemented subjects older than 50 years were included in the study, 581 recruited in the community, and 290 in the clinical setting. Participants in the clinical setting were more educated, had slightly lower MMSE scores, and presented more depressive symptoms than the participants in the community (Table 1).

**Figure 1:**
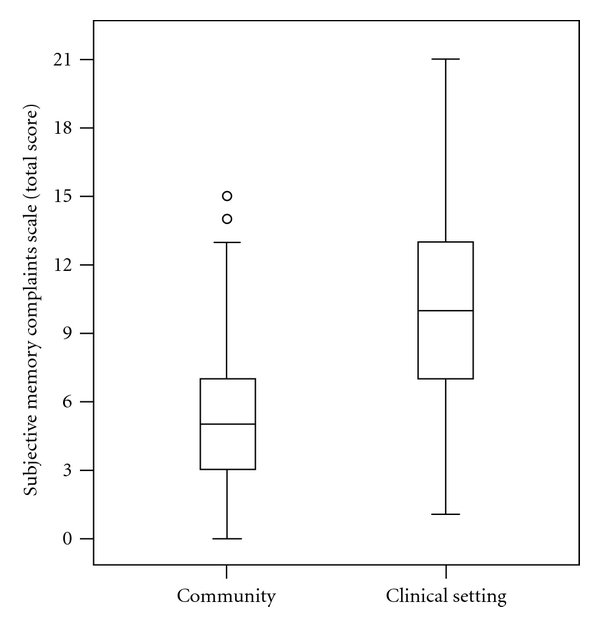
Total SMC scores in the community and in the clinical setting.

All participants in the clinical setting had complaints at least in one SMC item, and 20 (3.4%) participants in the community reported no memory complaints (total SMC score = 0). Since no differences in the total SMC scores were found among the different community settings (health itinerant unit, blood donor centre, leisure centre for retired people, and senior citizens college and university), the results from community participants were pooled together. Participants in the clinical setting had a higher total SMC score [10.3 ± 4.2 (1–21)] than those in the community [5.1 ± 3.0 (0–15)] (Figure 1), and this held true for almost all types of memory complaints (Table 2). 

The only exception was the SMC7 item, *Did you ever lose your way in neighbourhood?* few subjects reporting this difficulty, both in the clinical setting (4.8%) and in the community (3.1%).

**Figure 2:**
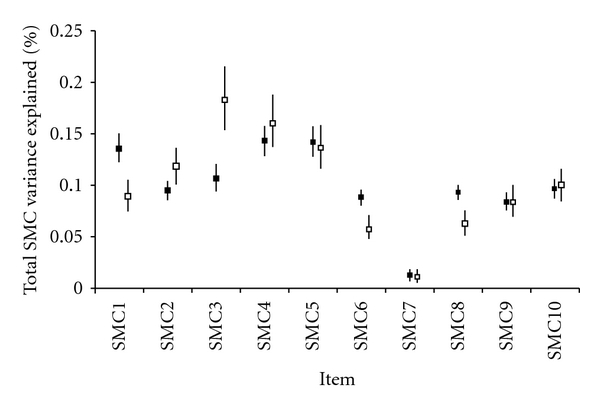
Percentage of the total subjective memory complaints scale (SMC) variance explained by each SMC item, controlling for depression score and education years. SMC: subjective memory complaints scale. *Eta-*squared values with 95% confidence intervals are shown. Black squares: community sample; open squares: clinical sample.

Analysing the weight of the different types of complaints to the global SMC score in the two groups, we found that SMC3, *Do you ever forget names of family members or friends? *contributed to only 11% of the total score variance in the community sample, and as much as 18% of the total score variance in the clinical sample (as shown in Figure 2, the 95% confidence intervals for the *eta-*squared values of SMC3 are separated). This was the item that contributed most to the total SMC score variance in the clinical group. In contrast, SMC1, which a general question about memory complaints, SMC6, *Do you ever have difficulties in finding particular words?,* and SMC 8, *Do you think more slowly than you used to?* contributed significantly less to the total SMC score in the clinical group.

## 4. Discussion

Elderly patients who seek for medical help in a memory clinic or hospital outpatient setting reported more prominent memory difficulties as compared to the subjects in the community. Nevertheless, only 20 community participants reported to be completely free from memory difficulties (i.e., to say, had 0 in the SMC total score). The participants in the clinical setting scored higher in almost all SMC items, reflecting more problems in different types of memory complaints. The exception was item SMC7, about being lost in the neighbourhood, to which few participants answered positively, as found in previous studies [5]. Difficulties in spatial orientation in known places may well reflect the beginning of a dementing disorder like Alzheimer's disease. In a recent study, the complaining of difficulties on finding one's way around familiar streets was highly associated with objective cognitive impairment [15].

The hypothesis advanced in the present work, that some types of memory complaints would be selectively reported by nondemented elderly subjects in the clinical setting, as compared to the community, was confirmed. Forgetting names of family members or friends contributed more strongly to the global subjective memory complaints in the clinical setting. This memory difficulty is probably perceived as particularly worrisome, or more likely to impact on close interpersonal relationships. It is interesting that it is not just the problem with names that is involved because subjects in the clinical setting did not report more difficulties in finding particular words (item 6). The trouble with such a trivial task as remembering proper names of close people appears particularly disturbing. It is interesting that the neuronal basis for processing familiar proper nouns is different from other names and quite widespread, involving both cerebral hemispheres [16]. The perception of this type of memory complaint as worrisome is certainly justified since 20% percent of patients with early Alzheimer's disease report forgetting the names of relatives [17].

Factors other than the memory complaints themselves may of course influence whether elderly people seek for clinical help or not. The clinical participants were more educated, possibly with more awareness of the implications of memory problems and an easier access to clinical care. They also had more depressive symptoms, which could indeed drive their concern about memory problems. An association between depressive symptoms and reporting of memory complaints has been consistently found (see, for instance, [18, 19]). The influence of personality characteristics on the emergence of memory complaints was also emphasised [20]. A recent study, comparing patients in a memory clinic and non-help-seekers, found that beliefs about memory, as well as the presence of a close relative with dementia, were associated with the decision to seek help [21].

It must be recognized that in the present study the evaluation of memory complaints was based on a single scale, the subjective memory complaints scale. Although this scale has items considered representative of common memory complaints [5], the results may not necessarily be generalizable to other instruments of memory complaints evaluation [22].

In conclusion, the clinical diagnosis of mild cognitive impairment relies on the presence of memory complaints in subjects who seek for medical help. The present study suggests that both the global severity of memory complaints and the type of memory difficulties reported, particularly forgetting names of family members or friends, are associated with the clinical setting. Further research should clarify the reasons why some elderly people seek for medical help, and others do not, since important consequences for the screening of early cognitive decline in the community may ensue.

## Figures and Tables

**Table 1:** Characteristics of the participants.

	Community	Clinical setting	Statistical significance
Number of participants (*n*)	581	290	
Age [years, mean ± SD (range)]	67.4 ± 9.0 (50–92)	67.6 ± 8.6 (50–88)	*P* = 0.679*
Gender (female/male)	355/226	163/127	*P* = 0.187^#^
Education [years, mean ± SD (range)]	6.1 ± 4.1 (0–19)	10.7 ± 4.5 (2–18)	*P* < 0.001*
MMSE [mean ± SD (range)]	28.0 ± 1.8 (23–30)	27.3 ± 2.0 (23–30)	*P* < 0.001*
GDS [mean ± SD (range)]	3.5 ± 2.9 (0–14)	5.2 ± 3.4 (0–15)	*P* < 0.001*

MMSE: mini-mental state examination; GDS: geriatric depression scale; *Student's *t* test; ^#^Fisher's exact test.

**Table 2:** Subjective memory complaints.

	Community	Clinical setting	Statistical significance*
	mean ± SD	mean ± SD	
(1) Do you have any complaints concerning your memory?	1.13 ± 0.70	2.05 ± 0.71	***P* < 0.001**
(2) Do other people find you forgetful?	0.36 ± 0.51	1.03 ± 0.69	***P* < 0.001**
(3) Do you ever forget names of family members or friends?	0.32 ± 0.54	1.15 ± 0.95	***P* < 0.001**
(4) Do you often forget where things are left?	0.78 ± 0.68	1.60 ± 0.91	***P* < 0.001**
(5) Do you often use notes to avoid forgetting things?	0.60 ± 0.61	1.22 ± 0.72	***P* < 0.001**
(6) Do you ever have difficulties in finding particular words?	0.48 ± 0.50	0.66 ± 0.48	***P* < 0.001**
(7) Did you ever lose your way in neighbourhood?	0.03 ± 0.17	0.05 ± 0.22	*P* = 0.201
(8) Do you think more slowly than you used to?	0.40 ± 0.49	0.84 ± 0.62	***P* < 0.001**
(9) Do your thoughts ever become confused?	0.45 ± 0.53	0.73 ± 0.67	***P* < 0.001**
(10) Do you have concentration problems?	0.52 ± 0.55	0.94 ± 0.70	***P* < 0.001**

Total SMC score	5.1 ± 3.0 (0–15)	10.3 ± 4.2 (1–21)	***P* < 0.001**

SMC: subjective memory complaints scale; *Mann-Whitney U-test

Scoring of items 1, 3, and 4: 0: no; 1: yes, but no problem; 2: yes, problem; 3: yes, serious problem.

Scoring of items 2 and 5: 0: no; 1: yes, sometimes; 2: yes, often.

Scoring of items 6 and 7: 0: no; 1: yes.

Scoring of items 8, 9 and 10: 0: no; 1: yes; 2: yes, serious problem.
